# Trends in Infertility Care Among Commercially Insured US Women During the COVID-19 Pandemic

**DOI:** 10.1001/jamanetworkopen.2021.28520

**Published:** 2021-10-06

**Authors:** Beth Zhou, Ammar Joudeh, Milli J. Desai, Brian Kwan, Vinit Nalawade, Brian W. Whitcomb, H. Irene Su

**Affiliations:** 1 Division of Reproductive Endocrinology and Infertility, Department of Obstetrics, Gynecology, and Reproductive Sciences, University of California San Diego, La Jolla; 2 Department of Obstetrics, Gynecology, and Reproductive Sciences, University of California San Diego, La Jolla; 3 Division of Biostatistics and Bioinformatics, Department of Family Medicine and Public Health, University of California San Diego, La Jolla; 4 Moores Cancer Center, University of California San Diego, La Jolla; 5 Department of Radiation Medicine and Applied Sciences, University of California San Diego, La Jolla; 6 Department of Biostatistics and Epidemiology, School of Public Health and Health Sciences, University of Massachusetts, Amherst; 7 OptumLabs Visiting Fellow, Eden Prairie, Minnesota

## Abstract

This cross-sectional study uses interrupted time series analysis of administrative claims data to evaluate trends in infertility and assisted reproductive technology utilization rates, including pattern differences by age, income, or race and ethnicity, among commercially insured US women during the COVID-19 pandemic.

## Introduction

Early in the COVID-19 pandemic, the American Society for Reproductive Medicine (ASRM) advised clinicians in March 2020 to suspend infertility care despite the time-sensitive nature of fertility. In April 2020, the ASRM recommended cautious continuation of care. Although the US birth rate has decreased during the pandemic,^1^ the magnitude, direction, and duration of change in infertility care is unknown. We evaluated trends in infertility and assisted reproductive technology (ART) service utilization rates during the pandemic and whether patterns differed by age, income, or race and ethnicity, given the differential effects of COVID-19 on socioeconomic groups^2^ and known socioeconomic disparities in infertility care.^3^

## Methods

For this cross-sectional study using an interrupted time series design, we used deidentified administrative claims data from the OptumLabs Data Warehouse to evaluate infertility service utilization rates among commercially insured US women aged 18 to 50 years between February 2018 to December 2020. The data warehouse contains information on more than 200 million patients covered by a US commercial health plan. Socioeconomic data were derived from public information for approximately 73% of enrollees.^4^ We used an interrupted time-series design^5^ with linear spline regression to evaluate the effects of the COVID-19 pandemic on infertility outcomes and estimated group effects overall and on rates of decline and recovery in the use of infertility and ART services, while accounting for time trends and confounding. We modeled claims for infertility (diagnosis or workup) and ART services (eTable in the Supplement) before the pandemic (February 2018 to February 2020), during care suspension (March to April 2020), and after care continuation (May to December 2020). Female age (18-34, 35-37, 38-40, 41-42, or 43-50 years), race and ethnicity (Asian, Black, Hispanic, White, or unknown), and household income (<$75 000, $75 000-$199 999, ≥$200 000, or unknown) were examined using interaction terms to assess differences in the decline and recovery in infertility and ART services utilization rates by group. Race and ethnicity were studied because of the differential effect of COVID-19 on racial and ethnic minority groups and known disparities in infertility care. The race and ethnicity data are derived from public information and estimates by a nationally recognized supplier of consumer marketing data.^4^ Statistical analysis was performed with R software (version 4.0.2; R Core Team), and *P* < .05 was the threshold for significance.

This study followed the Strengthening the Reporting of Observational Studies in Epidemiology (STROBE) reporting guideline. The study was exempt from institutional review board review because the use of deidentified data from the OptumLabs data set falls under exemptions specificed by the Common Rule.

## Results

**Figure 1.**
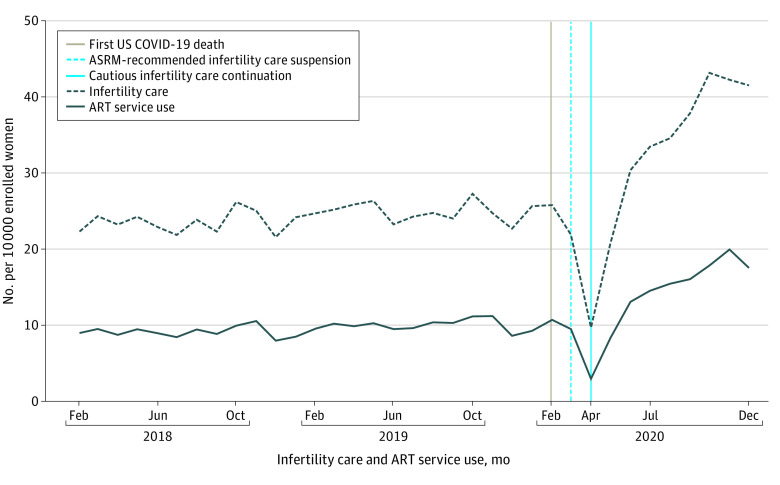
Trends in Utilization Rates of Infertility and Assisted Reproductive Technology Services Among Commercially Insured US Women Aged 18 to 50 Years From February 2018 to December 2020 ART indicates assisted reproductive technology; ASRM, American Society for Reproductive Medicine.

**Figure 2.**
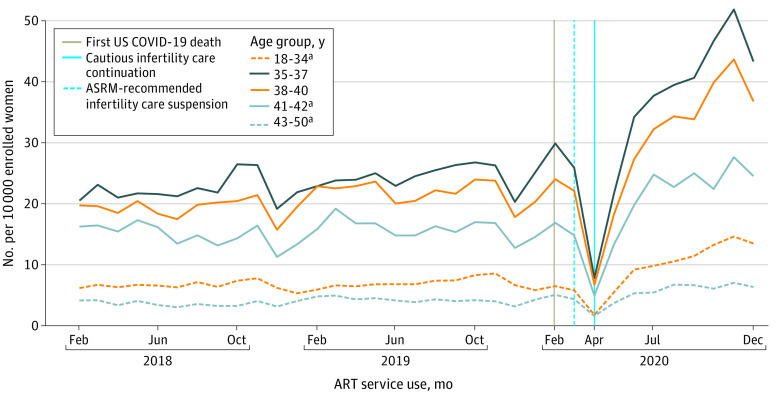
Trends in Utilization Rates of Assisted Reproductive Technology Services Among Commercially Insured US Women by Age ART indicates assisted reproductive technology; ASRM, American Society for Reproductive Medicine. ^a^*P* for interaction < .001 for ages 18 to 34 years and ages 43 to 50 years and *P* for interaction = .005 for ages 41 to 42 years compared with the recovery rate of the reference group (women ages 35-37).

The OptumLabs Data Warehouse claims data indicated that 8 755 271 women were eligible for this study. We observed incremental increases in infertility and ART service utilization rates before the pandemic (1.2 vs 0.72 per 10 000/y), sharp decreases during care suspension (−50.4 vs −21.6 per 10 000/y), and sharp recoveries after care continuation (96.0 vs 42.6 per 10 000/y) that were sustained through the end of the study (Figure 1). Patterns in infertility and ART services use were similar in all analyses. Before the pandemic, women aged 35 to 37 years had the highest ART utilization rate (29.8 per 10 000), followed by women aged 38 to 40 (24.0 per 10 000), 41 to 42 (16.8 per 10 000), 18 to 34 (6.4 per 10 000), and 43 to 50 (5.0 per 10 000) (Figure 2). The decline and recovery in utilization rates varied by age. Recovery rates adjusted for race and ethnicity and income were similar for women aged 35 to 37 and those aged 38 to 40 years (55.2 vs 46.8 per 10 000/y; *P* for interaction = .60), but they were significantly higher compared with women aged 18 to 34, 41 to 42, and 43 to 50 years (19.2, 28.8, and 8.4 per 10 000/y, respectively; *P* for interaction < .001 for ages 18 to 34 years and ages 43 to 50 years, *P* for interaction = .005 for ages 41 to 42 years). Before the COVID-19 pandemic, the ART utilization rate was highest among Asian women (19.1 per 10 000), followed by White, Black, and Hispanic women (9.2, 5.3, and 4.5 per 10 000, respectively; *P* < .001). The recovery rate after April 2020 was faster for Asian women compared with White women (105.6 vs 40.8 per 10 000/y; *P* for interaction < .001), whereas utilization rates were comparable among other racial and ethnic groups. The ART utilization rate before the COVID-19 pandemic was positively associated with income, but changes in utilization rates over time did not vary by group.

## Discussion

The results of this study indicate a rapid and sustained increase in infertility care and use of ART services after April 2020 that surpasses prepandemic levels and seemingly exceeds an unfulfilled need from the March 2020 care suspension period. These findings support the high priority placed on family building across age, socioeconomic status, and racial and ethnic groups despite potential study limitations. These limitations include the generalizability of a commercially insured population, the inability to restrict the study population to enrollees with infertility care benefits or examine enrollees by geographic region, and the potential misclassification of race and ethnicity. If these findings are reflected in the general US population, the record-low birth rates in December 2020 (from conception early in the pandemic) may reverse^1,6^ and an increasing demand for fertility care may challenge current care capacity. Lower utilization rates and slower recovery rates of infertility care among women older than 40 years raises concerns attributable to age-dependent fertility declines. Persistent disparities with Black race, Hispanic ethnicity, and lower income were observed but were not associated with differential recovery. Studies on the sustainment of increased infertility care use are needed.

## Notes

Supplement. **eTable.** Infertility Diagnosis and Assisted Reproductive Technology Treatment Administrative Claims Codes
eReferences
