# Supplementary material for: Trends in Infertility Care Among Commercially Insured US Women During the COVID-19 Pandemic
Source: JAMA Netw Open. 2021 Oct 6;4(10):e2128520. doi: 10.1001/jamanetworkopen.2021.28520 (PMC8495534; doi:10.1001/jamanetworkopen.2021.28520)
Supplement: Supplement. — eTable. Infertility Diagnosis and Assisted Reproductive Technology Treatment Administrative Claims Codes eReferences [file jamanetwopen-e2128520-s001.pdf]

Supplementary Online Content

Zhou B, Joudeh A, Desai MJ, et al. Trends in infertility care among commercially insured US women during the COVID-19 pandemic. *JAMA Netw Open*. 2021;4(10):e2128520. doi:10.1001/jamanetworkopen.2021.28520

eTable. Infertility Diagnosis and Assisted Reproductive Technology Treatment Administrative Claims Codes

eReferences

This supplementary material has been provided by the authors to give readers additional information about their work.

© 2021 Zhou B et al. *JAMA Network Open*.

eTable. Infertility Diagnosis and Assisted Reproductive Technology Treatment Administrative Claims Codes[1,2]

Code name	ICD-9	ICD-10	CPT	HCPCS
Infertility diagnosis and evaluation[a]	V26.2, V26.42, 257.1, 257.2, 606, 608.87, 608.89, 614.6, 628	E29.1, N46, N53.1, N97, Z31.41, Z31.62, Z31.81	58340, 58345, 58350, 74740, 74742	
Assisted reproductive technology[b]	V26.81, V26.82,	Z31.7, Z81.83, Z81.84	58970, 58974, 76948, 89250-89255, 89257, 89258, 89260, 89261, 89268, 89272, 89280, 89281, 89290, 89291, 89337, 89352, 89354, 89356, 89398, 00357T, 0058T	S0132, S4011, S4015-S4018, S4020-S4023, S4025, S4037

[a]Infertility and fertility preservation counseling, fertility testing, fallopian tube patency evaluation
[b]Oocyte retrieval, ART monitoring procedures and medications, ART laboratory procedures, cryopreservation, embryo transfer

eReferences

1. Coding Committee of the American Society for Reproductive M. Correct coding for laboratory procedures during assisted reproductive technology cycles. *Fertil Steril.* 2016;105(4):e5-e8.
2. Murugappan G, Li S, Lathi RB, Baker VL, Luke B, Eisenberg ML. Increased risk of severe maternal morbidity among infertile women: analysis of US claims data. *Am J Obstet Gynecol.* 2020;223(3):404 e401-404, e420.

© 2021 Zhou B et al. *JAMA Network Open*.
